# Hepatitis virus (HCV) diagnosis and access to treatment in a UK cohort

**DOI:** 10.1186/s12879-018-3367-3

**Published:** 2018-09-14

**Authors:** Emily Adland, Gerald Jesuthasan, Louise Downs, Victoria Wharton, Gemma Wilde, Anna L. McNaughton, Jane Collier, Eleanor Barnes, Paul Klenerman, Monique Andersson, Katie Jeffery, Philippa C. Matthews

**Affiliations:** 1 Department of Paediatrics, Peter Medawar Building for Pathogen Research, South Parks Road, Oxford, OX1 3SY UK; 2 Department of Infectious Diseases and Microbiology, John Radcliffe Hospital, Headley Way, Headington, Oxford, OX3 9DU UK; 3 Department of Hepatology, John Radcliffe Hospital, Headley Way, Headington, Oxford, OX3 9DU UK; 4 Nuffield Department of Medicine, Peter Medawar Building for Pathogen Research, South Parks Road, Oxford, OX1 3SY UK; 5 Oxford NIHR Biomedical Research Centre, John Radcliffe Hospital, Headley Way, Headington, Oxford, OX3 9DU UK

**Keywords:** HCV, Antigen, Antibody, Screening, Genotype, Epidemiology, Prison, Diagnosis, Ethnicity, DAA, Treatment, Cure, Sustainable development goals

## Abstract

**Background:**

As direct acting antiviral (DAA) therapy is progressively rolled out for patients with hepatitis C virus (HCV) infection, careful scrutiny of HCV epidemiology, diagnostic testing, and access to care is crucial to underpin improvements in delivery of treatment, with the ultimate goal of elimination.

**Methods:**

We retrospectively studied microbiology records from a large UK teaching hospital in order to compare the performance of HCV screening and diagnostic tests (antibody, antigen and HCV RNA detection). Having described a local cohort of adults with active HCV infection, we investigated the proportion who attended hospital appointments, were prescribed direct acting antiviral (DAA) therapy, and cleared HCV RNA following treatment.

**Results:**

Over a total time period of 33 months between 2013 and 2016, we tested 38,509 individuals for HCV infection and confirmed a new diagnosis of active HCV infection (HCV-Ag + and/or HCV RNA+) in 353 (positive rate 0.9%). Our in-house HCV-Ab screening test had a positive predictive value of 87% compared to repeat HCV-Ab testing in a reference laboratory, highlighting the potential for false positives to arise using this test. HCV-Ag had 100% positive predictive value compared to detection of HCV RNA. There was a strong correlation between quantitative HCV-Ag and HCV RNA viral load (*p* < 0.0001). Among the cases of infection, genotype-1 and genotype-3 predominated, the median age was 37 years, 84% were male, and 36% were in prison. Hepatology review was provided in 39%, and 22% received treatment. Among those who received DAA therapy with 12 weeks of follow-up, 93% achieved a sustained virologic response (SVR_12_).

**Conclusions:**

HCV-Ag performs well as a diagnostic test compared to PCR for HCV RNA. Active HCV infection is over-represented among men and in the prison population. DAA therapy is successful in those who receive it, but a minority of patients with a diagnosis of HCV infection access clinical care. Enhanced efforts are required to provide linkage to clinical care within high risk populations.

## Background

The World Health Organization (WHO) estimates that 71 million people are chronically infected with the Hepatitis C Virus (HCV), and 0.4 million people die each year as a consequence [1, 2]. International targets have been set for the elimination of viral hepatitis as a public health threat by the year 2030 [2, 3], underscoring an urgent need for improved case-finding. The need for enhancing HCV diagnosis has also become more pertinent as a result of the increasing availability and success of Direct Acting Antiviral (DAA) treatment [4–7]. Globally, only 15–20% of individuals with chronic HCV infection are currently thought to be aware of their diagnosis, with even fewer receiving treatment [5, 8, 9].

Streamlined, accurate and accessible HCV diagnosis is important not only as a pathway to treatment for individual patients, but also to allow confident estimates of the true prevalence of chronic HCV infection in different settings. Epidemiologic data are crucial to underpin appropriate allocation of resources and development of infra-structure for treatment [10]. Screening and diagnosis of HCV infection is based on three different approaches, which may be used alone or in combination. These are (i) detection of an IgG antibody by ELISA (HCV-Ab); (ii) detection of HCV core antigen (HCV-Ag); (iii) Nucleic acid testing (NAT) to detect HCV RNA by PCR (Table 1). Of these, only (ii) and (iii) can confirm active infection.

**Table 1:** Comparison of diagnostic laboratory tests used to detect exposure and activity of HCV infection

Screening tool	HCV-Ab	HCV-Ag	PCR for HCV RNA
Benefits	♦ Widely available; ♦ Inexpensive; ♦ Much experience and data for use as first-line approach to screening for HCV exposure (underpins many old seroprevalence studies).	♦ Diagnostic of active infection (not past exposure); ♦ Improved specificity and reduced window period compared to HCV-Ab [14, 29, 42–45].	♦ Accepted gold-standard diagnostic test for active infection (not past exposure); ♦ Allows quantitative monitoring of viraemia; useful for monitoring therapy; ♦ Genome amplification allows other information to be ascertained (e.g. genotype; drug resistance); ♦ Can potentially be applied to dried blood spots (DBS).
Challenges	♦ Subject to inter-assay variability and a variable rate of false positive results [46, 47]; false positive has been associated with ethnicity [48, 49], age [48], raised IgM and erythrocyte sedimentation rate (ESR) [46], auto-antibodies [50], and prosthetic devices [51]; ♦ Test of exposure, not of active infection, so should be followed up with a more specific diagnostic test.	♦ Not universally available; ♦ More expensive than HCV-Ab; ♦ Not consistently regarded as sufficiently sensitive to replace PCR.	♦ Not universally available; ♦ Expensive: beyond the financial reach of many resource-limited settings.

Reliance upon HCV-Ab screening has potentially distorted epidemiological data upon which resource-planning depends [11], as this approach includes detection of individuals who have cleared infection either spontaneously or through treatment (estimating exposure as well as active infection), and also includes false positives. As a result, there has been a progressive move towards using HCV-Ag and/or HCV PCR to determine accurately the population prevalence of active infection [1, 12, 13]. Although sensitivity and specificity of HCV-Ag testing appears to perform well when compared head-to-head with PCR [10, 13, 14], there are still potential doubts over whether this test is sufficiently sensitive to be widely implemented as a primary screening tool, and recent WHO guidelines continue to recommend use of the HCV-Ab test for first line screening [12]. A careful balance must be struck between managing cost and optimising specificity without sacrificing sensitivity [15–18].

We here set out to assess our progress in diagnosing and treating HCV infection in a tertiary referral UK hospital. We reviewed the performance of our local HCV testing protocol in two different time periods, first when screening was undertaken using an HCV-Ab test only, and subsequently following the introduction of a combined approach using HCV-Ab screening and HCV-Ag diagnostic confirmation. In each period, we went on to evaluate further using PCR for HCV RNA. Collating these data allowed us to evaluate the performance of different screening and diagnostic tests, to describe the characteristics of our local cohort, and to determine the proportion of those with active HCV infection who attend a hepatology clinic and receive treatment.

## Methods

### Setting and cohort

Our microbiology laboratory in the South East of the UK is located in a large tertiary referral teaching hospital (http://www.ouh.nhs.uk/) that provides one million patient contacts a year, and handles samples referred from the community as well as four in-patient sites.

**Fig. 1:**
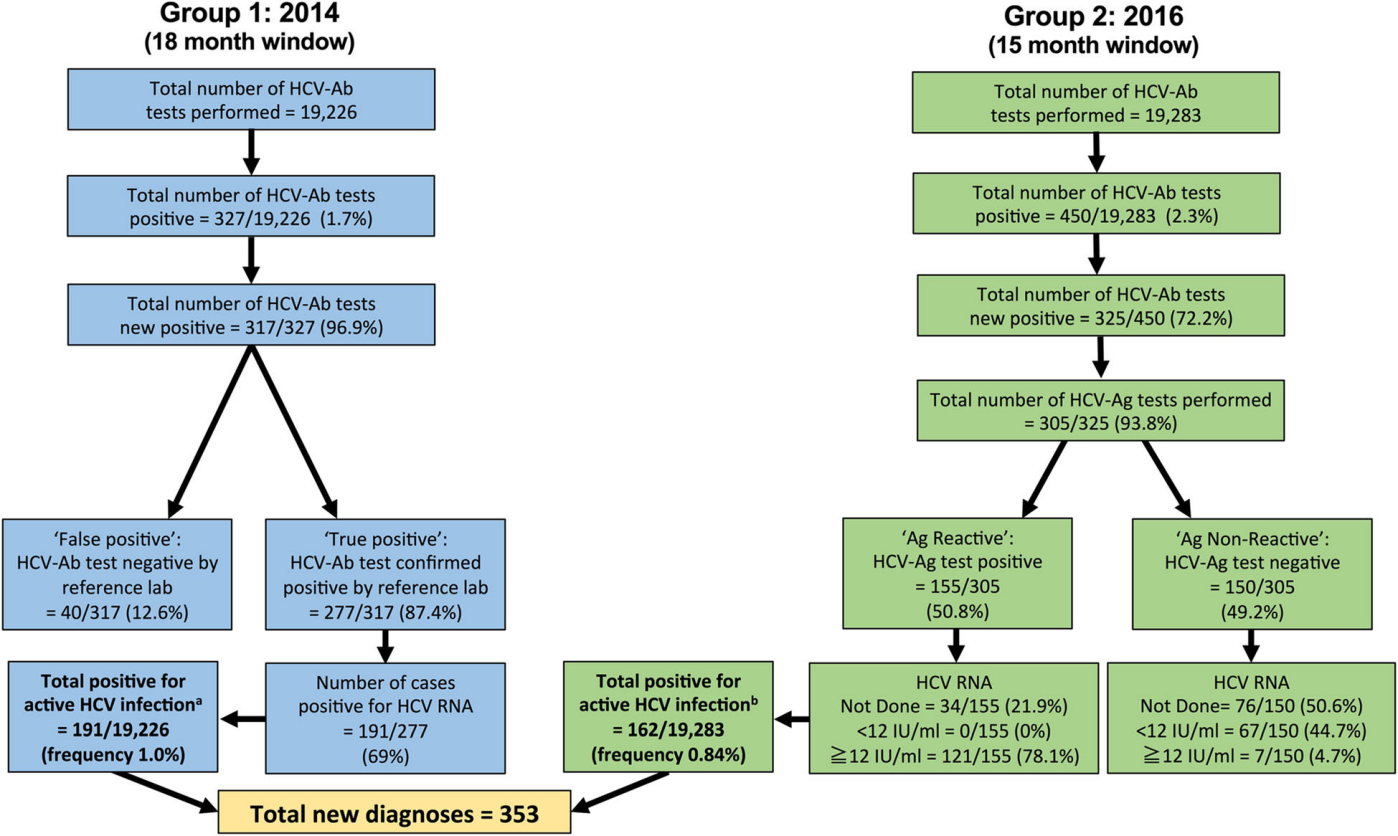
Algorithms describing HCV screening and diagnosis in a UK teaching hospital laboratory in 2014 (Group 1) and 2016 (Group 2). ^a^The total positive rate for Group 1 is defined as the number of samples that were HCV RNA positive (*n* = 191) divided by the total number of samples screened (n = 19,226). ^b^The total positive rate for Group 2 is defined as samples that were deemed positive for active HCV infection based on interpretation of combined results (this includes HCV-Ag positive and not further screened (n = 34), plus any sample that was HCV-RNA positive irrespective of the HCV-Ag result (*n* = 128, comprising 121 HCV-Ag positive, and 7 HCV-Ag negative samples), divided by the total number of samples screened (*n* = 19,283). The lower limit of quantification for HCV RNA was 12 iU/ml. There was no case of detectable HCV RNA below the limit of quantification. Full metadata for this cohort can be found in the supporting data-file: 10.6084/m9.figshare.5355097

We retrospectively interrogated electronic microbiology records for all HCV assays performed within two defined time-intervals, during which different diagnostic algorithms were operating, in each case starting with a screening test and then proceeding to confirm active infection. These are summarized in Fig. 1 and outlined as follows:

i. **Group 1** (18 months; January 2013–June 2014). Samples were screened for HCV-Ab using an ADVIA Centaur automated immunoassay (Bayer). HCV-Ab-positive samples (excluding repeat samples from patients with a pre-existing HCV diagnosis) were sent for confirmatory testing by the regional reference laboratory (Public Health England, Colindale), using two further ELISA tests (Ortho and BioRad). Antibody positive samples (based on sample:cut-off ratio > 1) were tested for HCV RNA.
ii. **Group 2** (15 months; January 2015–March 2016). HCV testing was undertaken using a combination of HCV-Ab and HCV-Ag, using Abbott Architect i2000SR, with Diasorin Liason XL for confirmation of HCV-Ab. The HCV-Ag assay provides a quantitative result up to an upper limit of 20,000 fmol/L. We classified values of ≥3 fmol/L as positive (as previously reported [19]). When we introduced the HCV-Ag assay, we tested the same sample for HCV RNA in parallel, for the puporses of validation. Following this validation period, we stopped routinely testing the same sample for HCV RNA, and changed the protocol to request a second sample for RNA testing (only in those that are HCV-Ag positive). This allows us to confirm the diagnosis using two separate samples, in keeping with good laboratory practice, and also provides material for genotyping.

For Groups 1 and 2, samples were tested for HCV RNA using the Abbott HCV M2000 assay.

Some individuals were tested for HCV infection on > 1 occasion; we removed duplicate tests from our overall positive cohort using unique identifiers (hospital number or NHS number). We recorded patient age, sex, and the location from which the sample was sent. Treatment data were captured and recorded from an electronic database within the Hepatology Department. Response to treatment was defined as sustained virologic response (undetectable HCV RNA using PCR) at ≥12 weeks following the end of therapy (SVR_12_).

Ethnic origin is not routinely captured data in hospital electronic systems. Prior to anonymisation, we therefore used an analytical tool to estimate ethnicity, applying Onolytics software for all patients for whom a full name was part of the electronic record (https://onolytics.com [20–22]). This software was developed in 2006–7 funded by Economic and Social Research Council (ESRC) Knowledge Transfer Partnerships, and sets out to determine probable ethnic origin based on name.

### Data analysis

Statistical analysis was performed using GraphPad Prism v.7.0b and Googlesheets (https://docs.google.com/spreadsheets). We compared binary values using Fisher’s Exact Test, Mann-Whitney U test for continuous non-parametric data, chi-square for analysis of categorical data, and linear regression for correlation between continuous variables.

### Ethics approval

Ethics approval was not required, as this study was undertaken as a departmental quality improvement exercise within microbiology using anonymised patient data, and completed the audit cycle for previously approved audit projects [23, 24]. Data for Onolytics analysis were handled separately, subject to a confidential disclosure agreement drawn up by University of Oxford Research Services (February 2016).

## Results

### HCV testing: Frequency and characteristics of infection

In total, we present data for 38,509 HCV tests done during the two intervals reviewed. On average we performed an average of 1068 tests / month during the earlier phase of the study (Group 1) and 1286 tests / month in the later time period (Group 2); Fig. 1.

We identified 353 active HCV infections across Group 1 and Group 2, using a combination of HCV-Ag and/or HCV RNA testing. We estimated the frequency of active HCV infection within this cohort at 0.9% based on a combined numerator (*n* = 353), and using the total number of samples tested as the denominator (*n* = 38,509); Fig. 1. Comparing the two time periods, there was no change in the frequency of positive HCV-Ab testing: HCV-Ab frequency 317 / 19,226 (1.6% in Group 1) vs. 325 / 19,283 (1.7% in Group 2); *p* = 0.8, chi-sqaure test. There was also no significant difference in the frequency of confirmed active HCV infection (based on HCV-Ag and/or HCV RNA PCR): 191 / 19,226 (1.0% in Group 1) vs. 162 /19,283 (0.8% in Group 2); *p* = 0.13, chi-square test.

**Fig. 2:**
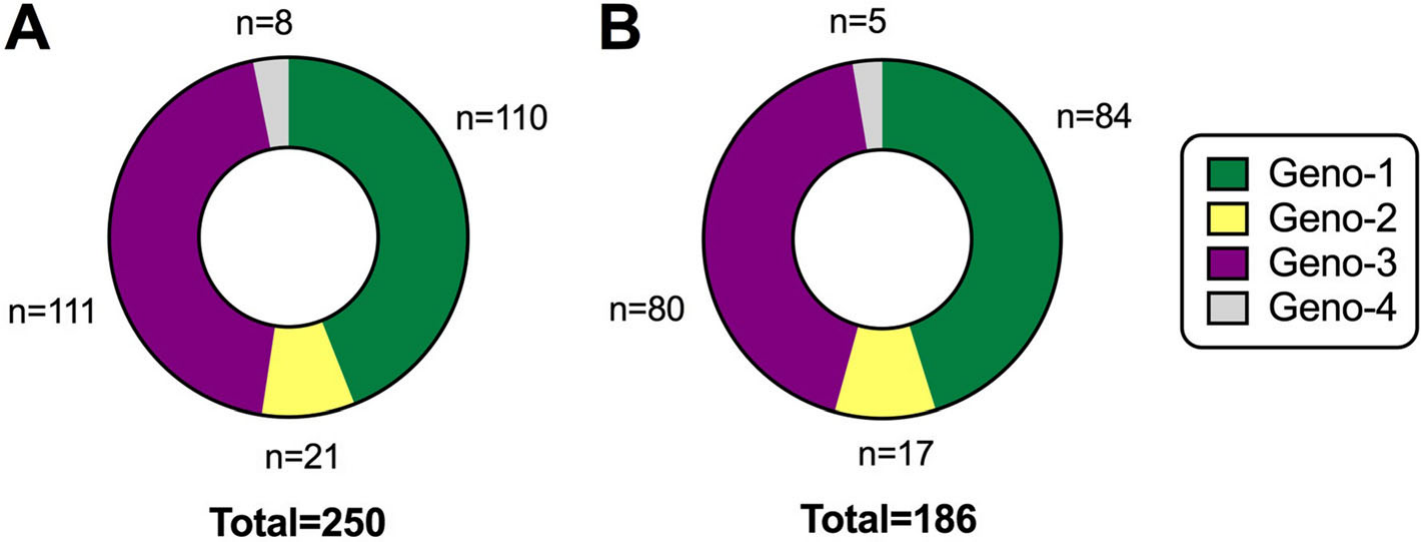
Distribution of HCV genotypes in a UK cohort. **a** Data for an extended cohort of 250 individuals for whom HCV genotyping was undertaken in our laboratory between 2014 and 2016 (includes the 186 individuals represented in panel **b**, plus an additional 64 individuals who had genotyping undertaken within this time period but were not captured within Group 1 or Group 2). **b** Data for 186 individuals for whom genotype was determined from among the cohort of 353 new HCV diagnoses within Group 1 and Group 2 of this study. There was no enrichment of a specific genotype in the prison population (prison population accounted for 30/84 geno-1 infections, and 34/80 geno-3 infections; *p* = 0.4 Fisher’s Exact Test)

Characteristics of the 353 individuals with active HCV infection are summarised in Table 2, and the complete metadata are available on-line (10.6084/m9.figshare.5355097). The median age was 37 years (IQR 31–48). Men accounted for 55% of all individuals tested, but 84% of all new diagnoses (Table 2) [23]. Over one-third (36%) of new diagnoses were made in prison. Genotype was available in 186 cases (53% of new diagnoses), with genotype 1 (*n* = 84) and genotype 3 (*n* = 80) accounting for the majority (45% and 43%, respectively); Fig. 2.

**Table 2:** Characteristics of individuals screened for HCV infection in a UK teaching hospital in two time windows between 2014 and 2016

	Group 1 (2014)	Group 2 (2016)	Group 1 + Group 2 (2014–2016)
Total number HCV-Ab positive	317	325	642
Total number confirmed positive for active HCV infection (HCV-Ag/HCV RNA positive)	191	162	353
Number male (% of active infections)^a,b^	165 (86.3%)	132 (81.5%)	297 (84.1%)
Age in years (median and IQR)^a^	39 (31–49)	36 (30–46)	37 (31–48)
Location^a, c^			
- Primary Care	64 (33.5%)	46 (28.4%)	110 (31.2%)
- Hospital out-patient	17 (8.9%)	17 (5.2%)	34 (9.6%)
- Prison	61 (31.9%)	66 (40.7%)	127 (35.9%)
- Hospital in-patient^d^	22 (11.5%)	9 (5.5%)	31 (8.8%)
- Emergency Dept.	5 (2.6%)	6 (3.7%)	11 (3.1%)
- Sexual health clinic	16 (8.4%)	13 (8.0%)	29 (8.2%)
- Occupational health	2 (1.0%)	1 (0.6%)	3 (0.8%)
- Other locations^e^	4 (2.1%)	4 (2.5%)	8 (2.3%)
Ethnic origin^a^			
- Black	5 (2.6%)	1 (0.6%)	6 (1.7%)
- Asian	17 (8.9%)	7 (4.3%)	24 (6.8%)
- European	149 (78.0%)	100 (61.7%)	249 (70.5%)
- Unknown	20 (10.5%)	54 (33.3%)	74 (21.0%)

^a^Breakdown of characteristics is shown for the individuals who have confirmed active HCV infection, based on HCV-Ag and/or HCV RNA PCR

^b^Numbers of positive tests are shown with percentage of positive tests in brackets

^c^We did not have access to data for PWID, but prison location may be an important surrogate marker for this group

^d^Hospital in-patient locations include general surgery, orthopaedics, transplant, renal, haematology, intensive care, unspecified ward locations in the hospital, and in-patients in community hospitals

^e^Other locations include paediatrics, psychiatry, fertility and pathology

### Outcomes and performance of HCV-ab and HCV-ag assays

**Fig. 3:**
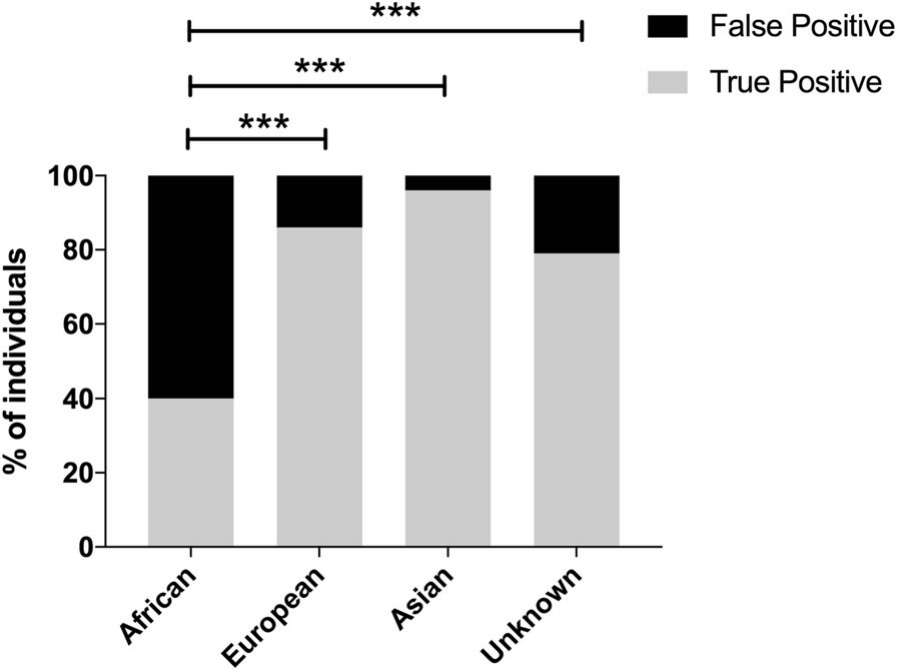
False positive HCV IgG antibody results according to ethnic origin in a UK cohort. Ethnicity was estimated using Onolytics software [26, 27]. Data shown are for a cohort recruited starting in 2014 (designated Group 1), screened using an in-house HCV-Ab (ADVIA Centaur automated immunoassay; Bayer) and confirmed using two further ELISA tests (Ortho and BioRad). ‘False positives’ are defined as those screening positive on ADVIA but subsequently negative, ‘true positives’ are defined as samples positive on all three tests. *P*-values obtained by Fishers Exact Test; *** *p* < 0.0005

In the earlier testing period (Group 1), 277/317 HCV-Ab positive samples were positive on confirmatory testing for HCV-Ab at the reference laboratory (Fig. 1), giving our in-house test a positive predictive value (PPV) of 87.4% compared to a regionally accepted standard. We used these results to investigate whether any host factors were associated with false positive antibody tests, and found that individuals identified as African have a higher chance of a false-positive HCV Ab test (Fig. 3). We confirmed this result by multivariate logistic regression analysis, in which African ethnicity was significantly associated with a false positive Ab test result (*p* = 0.0004), but age > 60 years and sex were not. Prison location was associated with a true positive Ab-test result (*p* = 0.01). We did not have access to data on representation of PWID in this cohort, but recognise that prison location is a likely surrogate marker for this risk factor for infection.

In the later testing period (Group 2), the PPV of the combined use of HCV-Ab plus HCV-Ag was 100% when compared to a gold-standard diagnostic test using PCR (Table 3).

**Table 3:** Outcome of diagnostic testing for HCV infection using core antigen detection (HCV-Ag) compared to gold standard PCR for HCV RNA

Outcome	Result (n)*
True positive	121
False negative	7
False positive	0
True negative	67
Test characteristic	Result (%)
Sensitivity	94.5
Specificity	100
Positive predictive value	100
Negative predictive value	90.5

**Fig. 4:**
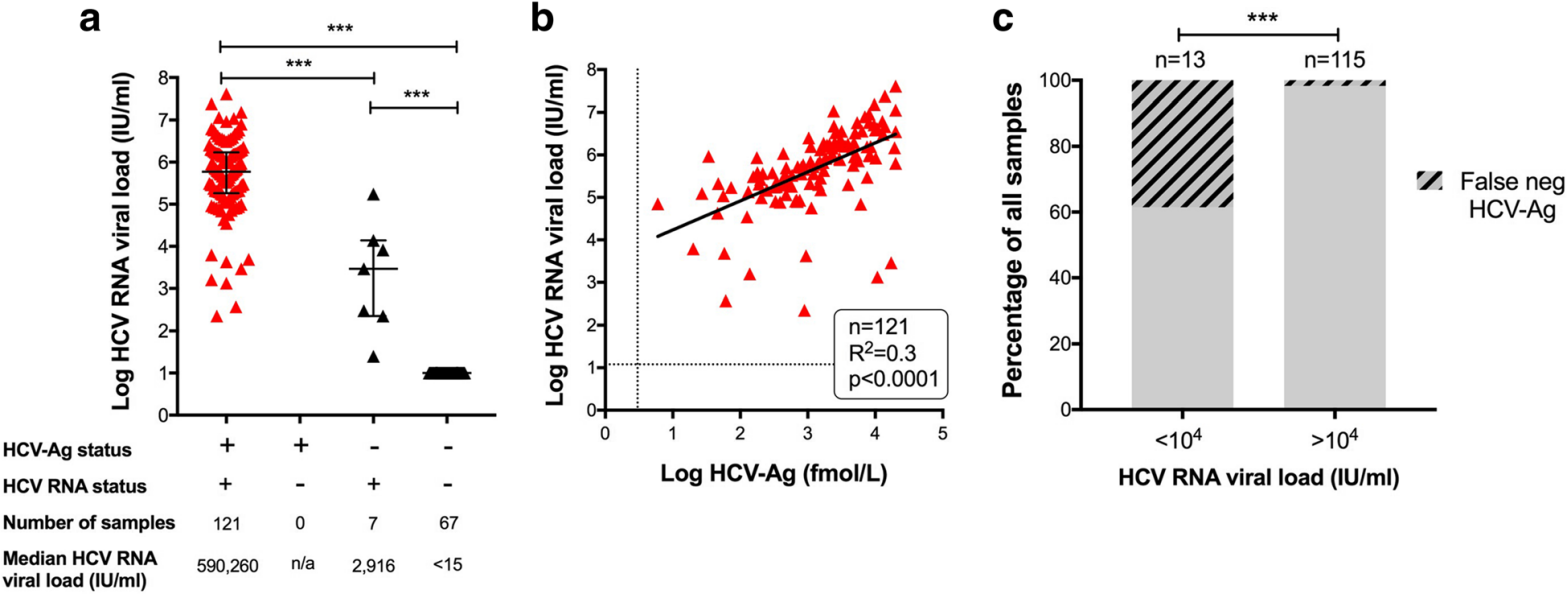
Relationship between HCV Antigen test and quantitative PCR for HCV RNA viral load. **a** Range of HCV RNA viral loads for samples testing HCV-Ag positive (*n* = 121) and HCV-Ag negative (*n* = 74). Median and interquartile range shown. *P*-values by Mann-Whitney U test; ***p < 0.0001. **b** Relationship between between HCV-Ag and HCV RNA viral load for all samples testing HCV-Ag positive (n = 121). Dashed lines represent threshold for detection for HCV RNA (12 IU/ml) and HCV-Ag (3 fmol/L). Solid line represents linear regression analysis; R^2^ = 0.3, *p* < 0.0001. **c** Percentage of samples testing false-negative for HCV-Ag according to HCV RNA viral load. *P*-value by Fisher’s Exact test ***p < 0.0001. Data are shown for 128 samples for which both HCV RNA and HCV-Ag testing was undertaken. HCV-Ag was falsely negative (< 3 fmol/L) in 5/13 cases with HCV RNA < 10^4^ IU/ml, and in 2/115 cases with HCV RNA > 10^4^ IU/ml

*Results pertain to all HCV-antibody individuals in ‘Group 2’, based on samples that were tested with assays for both HCV-Ag and HCV RNA, defined as follows: true positives (HCV-Ag + and HCV RNA+); false negatives (HCV Ag- and HCV RNA+); false positives (HCV Ag + and HCV RNA-); true negatives (HCV Ag- and HCV RNA-). These groups are also shown in Fig. 4a. Threshold for positive HCV-Ag was 3 fmol/L

Individuals with a positive HCV-Ag test had a median HCV viral load of 5.9 × 10^5^ IU/ml (Fig. 4a), and there was a significant positive correlation between quantitative antigenaemia and viral load (r^2^ = 0.3, *p* < 0.0001; Fig. 4b). However, in a small proportion of cases, the HCV-Ag test was falsely negative (Table 4).

**Table 4:** Summary of seven adults in whom HCV core antigen (HCV-Ag) assay was falsely negative, as compared to PCR for HCV RNA as a gold-standard reference test

Age group (years)	Sex	Patient location	Ethnicity	HIV status	HCV Ag (fmol/L)	HCV Ab (sample/cut-off ratio)	Genotype	HCV viral load (IU/ml)
30–39	F	Sexual health	Unknown	negative	0.0	11.8	N/A	25
40–49	M	Primary care	European	negative	0.0	12.2	N/A	226
50–59	F	Hospital in-patient (General Medicine)	European	positive	0.0	3.1	N/A	302
20–29	M	Prison	European	negative	0.62	14.2	2b	2916
20–29	M	Prison	European	N/A	0.00	12.6	N/A	8232
30–39	F	Hospital out-patient	European	N/A	1.98	15.8	1b	13,860
30–39	M	Prison	European	negative	0.00	12.2	3a	174,834

N/A = not available. Total number of HCV-Ag tests carried out in this period *n* = 305. None of the patients with a false negative result underwent a repeat Ag test so laboratory error cannot be ruled out in this instance. Threshold for positive HCV-Ag defined as ≥3 fmol/L. Samples are ranked in ascending viral load order

Based on the strong correlation between HCV-Ag and viral load (Fig. 4b), and on a previous analysis that documents HCV-Ag down to a level equivalent to viral load 3000 IU/ml [13], we sought to determine whether the false negative HCV-Ag tests (*n* = 7) were associated with low viraemia. Indeed, in 5/7 cases, HCV RNA was < 10^4^ IU/ml, one of which had HCV RNA as low as 25 IU/ml. Among 128 samples for which we had both an HCV-Ag and HCV RNA assay result, the false-negative tests were significantly enriched in the group with HCV RNA < 10^4^ IU/ml (p < 0.0001, Fig. 4c).

### Clinical management and outcomes

Of 353 patients with a new HCV diagnosis, 142 (40%) attended a hepatology clinic appointment, 79 were treated (22% of the cohort) and 66 met the SVR_12_ criterion (19% of the whole cohort; 84% of all those treated). Among those treated with a DAA-based regimen (*n* = 59), a treatment endpoint was documented in 54, of which 50 were classified as SVR_12_ (93%). For individual treatment regimens, see full clinical metadata available on-line (10.6084/m9.figshare.5355097).

This study was not designed to examine or report on the outcomes of treatment. However, we examined existing treatment data to look for evidence of different outcomes between genotypes 1 and 3. Among treated genotype 1 infections with outcome data (*n* = 34), we recorded 32 cases of SVR_12_, and two cases of relapse. For treated genotype 3 (*n* = 30), there were 25 cases of SVR_12_ and five relapses, but this difference did not reach statistical significance (relapse rate 2/34 (6%) for geno-1 vs. 5/30 (17%) for geno-3; *p* = 0.2, Fisher’s Exact Test).

## Discussion

### Summary comments

Careful scrutiny of HCV screening, diagnosis and treatment is important so that enhanced efforts can be made to identify individuals with active infection in order to provide access to DAA therapy, and to move towards international elimination goals [3, 9, 13].

In our setting in the UK, 0.9% of all samples submitted for HCV testing had evidence of active HCV infection. Following the implementation of HCV-Ag testing as part of the diagnostic algorithm, the PPV of a positive test increased to 100%, slightly exceeding that reported by other recently published studies [14]. The close correlation between HCV-Ag and HCV RNA viral load suggests that, in the absence of having access to a quantitative PCR result, HCV-Ag may be a useful surrogate marker of viraemia, particularly at higher viral loads (e.g. if HCV RNA > 10^4^ IU/ml). Genotypes 1 and 3 predominated (in keeping with other published studies in this setting [25, 26]).

**Fig. 5:**
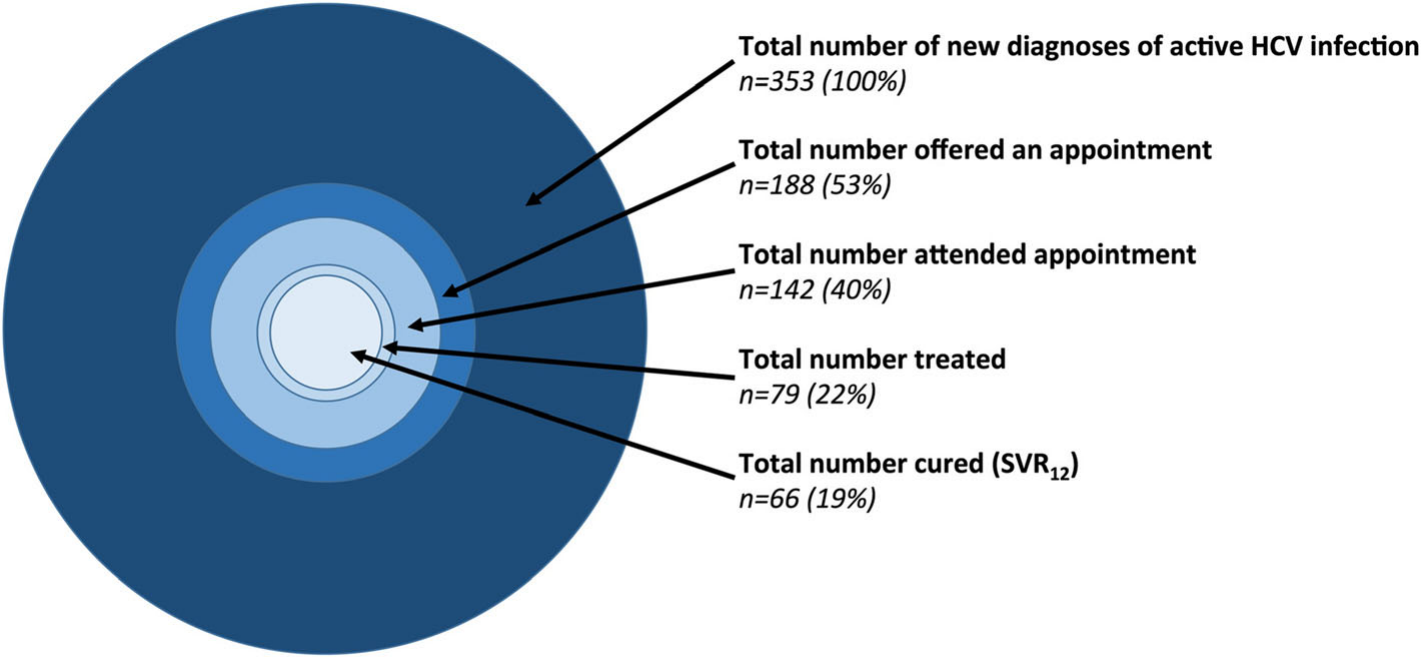
Graphical representation of the disparity between the number of individuals diagnosed with active HCV infection and those who access clinical review, treatment, and achieve SVR_12_. Summary of outcomes for the entire cohort is shown in Table 5. The percentages quoted in this figure represent the proportion of patients in each category from the total denominator of 353. Individuals diagnosed in prison were significantly less likely to attain an SVR_12_ endpoint (SVR_12_ was documented for 5/127 individuals in prison vs. 61/226 not in prison, *p* < 0.0001, Fisher’s Exact test)

Our data confirm the success of DAA treatment, with SVR_12_ documented in 93%. However, this cohort also highlights the substantial loss of patients at each step of the clinical ‘cascade’ (Fig. 5; Table 5). This is due to a combination of factors that include poor linkage between services (community, prisons, sexual health and secondary care), itinerant populations, specific challenges in providing follow-up for those in prison and PWID, and deaths.

**Table 5:** Summary of clinical care outcomes in 353 individuals with a diagnosis of chronic HCV infection

Treatment Status	Patient Classification	Number	Percentage of treatment subgroup^a^	Percentage of total cohort
Not yet treated (*n* = 130)	Offered appointment but did not attend	46	35.4	13.0
	Seen in another clinic (sexual health, prison, paediatrics)	32	24.6	9.1
	Seen by hepatology but not on treatment waiting list	23	17.7	6.5
	Seen by hepatology and on treatment waiting list for DAA	3	2.3	0.9
	Died	11	8.5	3.1
	Transferred out of area	10	7.7	2.8
	Seen in clinic but lost to follow-up	2	1.5	0.6
	Spontaneous clearer	3	2.3	0.8
Treated with DAA (*n* = 59)	SVR_12_	50	84.7	14.2
	Relapsed	4	6.8	1.1
	Outcome data pending	5	8.5	1.4
Treated with IFN/RBV (*n* = 20)	SVR_12_	16	80.0	4.5
	Relapsed	3	15.0	0.8
	No outcome data	1	5.0	0.3
Unknown (*n* = 150)	Not known to local services	144	100	40.8
TOTAL		353	400	100

^a^Treatment subgroup is defined as the four categories listed in the first column of this table. The total of this column is 300%, as the total for each of three subgroups is 100%

*DAA* = direct acting antivirals, *IFN* = interferon, *RBV* = ribavirin; SVR_12_ = sustained viraemic response at ≥12 weeks following therapy

### Relevance to laboratory and clinical practice

Although HCV-Ag testing can potentially replace a nucleic acid test for HCV diagnosis or monitoring in some settings [14, 27, 28], guidelines from the UK [29], North America [30] and the WHO [12] still advocate use of PCR as a definitive test following HCV-Ab (± HCV-Ag) screening. RNA PCR also remains the gold-standard approach to monitoring progress during and after treatment and is currently still recommended for genotyping, to underpin optimum choice of DAA regimen [31]. However, as we move towards pan-genotype treatments for HCV, a single diagnostic test, with the potential to be applied at the point-of-care, is an appealing strategy [13].

The small proportion of all diagnosed patients who access clinical care and receive successful treatment is in keeping with that reported in other centres [5, 32], reflecting many challenges for HCV elimination. Our data highlight the particular vulnerability of the prison population, in keeping with a worldwide estimate of 15% HCV prevalence in prisoners [33]. Offering treatment within the prison system has now become a realistic possibility, on the basis of oral DAA therapy, shortened treatment regimens, and a low rate of side-effects [34, 35].

In the longer term, bigger datasets are required to improve our insights into the HCV-infected population [32], and health economics analyses should be used to support optimum deployment of resources in different settings [36, 37]. On the global stage, optimization of laboratory testing and reduction of costs are essential to improve access to accurate diagnosis, advocacy for better testing and treatment for populations in resource-limited settings, and especially for targeting interventions at high-risk populations including MSM, PWID and prisons.

### Performance of HCV-ag test

Our calculated PPV of 100% for the HCV-Ag test exceeds a previous estimation of the PPV of the Ag test (94.7%) calculated from assimilation of data from other comparable reports [14]. However, PPV is dependent on the overall prevalence of infection, and will therefore vary between settings.

A recent meta-analysis concludes that the HCV-Ag test performs similarly to HCV RNA PCR when viral load is > 3000 iu/ml [13]. Thus this diagnostic tool provides a high sensitivity without unduly influencing specificity. The HCV-Ag assay is likely to perform less reliably at low viral loads, and a low threshold should be applied for using NAT to confirm a diagnosis in the settting of an equivocal HCV-Ag result (3–10 fmol/L). Another potential explanation for false negative HCV-Ag test is mutations in the core region of the HCV genome which could account for a failure of antigen detection [38], or potentially cause lack of PCR amplification if mutations occur in primer binding regions.

### Caveats and limitations

Our analysis must be set in the overall context of the low prevalence of HCV in our setting, and the retrospective approach to data collection. We cannot comment on population level epidemiology, as a large pool of individuals who are HCV-infected never have a screening or diagnostic test [9]. Due to gaps in the laboratory data-set, in which not every sample was tested for HCV RNA, we adopted a pragmatic definition of active HCV infection based on the best laboratory evidence available (in some cases this was an assay for HCV-Ag without an accompanying NAT). Our analysis of sensitivity and specificity of HCV-Ag testing could only be assessed in the subset of samples that had undergone testing with assays for both HCV-Ag and HCV RNA quantification.

The rate of false negative HCV tests is our population is likely to be low, but quantifying this was not possible within this study, as we relied on identifying samples that initially tested positive. In order to ascertain the PPV of the HCV-Ab test in-house, we referred to a Reference Laboratory test as ‘gold standard’. However, this repeat testing in a Reference Laboratory setting is itself subject to an error rate, and therefore may lead to a misrepresentation of our overall assay performance.

We found evidence that the HCV-Ab test performs poorly in individuals predicted to be of African origin. A similar high rate of false positive tests has previously been reported from Polynesia [39]. This illustrates how tests that have been validated in white European/Caucasian populations cannot necessarily be robustly applied in other settings. Although the tools used here have been validated [21, 22], use of name is an imperfect way to derive ethnic origin and is potentially confounded by a variety of factors, the most obvious of which is individuals who change their name through marriage.

In our setting, the sexual health clinic anonymises patient data, preventing robust linkage between services. We are therefore unable to trace outcomes for patients who were diagnosed via this route (8% of the total; Table 2). Likewise, consistent identification and tracing of individuals who use drugs and/or are in prison is challenging, and we cannot exclude the possibility of duplication of some of these individuals within our dataset.

In the context of this study we do not have prospective socio-demographic data that are required to investigate the reasons for the male excess we describe (e.g. MSM, PWID). Sex differences could also be accounted for by genuine biological discrepancies in susceptibility to, and outcomes of, infection between males and females [40, 41]. We have also not been able to characterise the prison population in detail; this group is difficult to treat and to follow-up, and so is at risk of worse outcomes, but we may have underestimated SVR_12_ due to missing data.

## Conclusions

A sensitive, affordable point-of care test in the form of an HCV-Ag test is a desirable solution for HCV diagnosis in many settings, and we have shown this to be reliable in most cases, although with a reduced sensitivity in the context of HCV viral loads < 10^4^ IU/ml. In concordance with other studies [5], our data highlight the ongoing need for multilateral efforts to provide access to diagnosis and routes into treatment, if success is to be achieved in the global targets for elimination of this infection as a threat to public health.

## Abbreviations

DAA: Direct acting antiviral
ELISA: Enzyme linked immunosorbent assay
HCV RNA: Hepatitis C ribonucleic acid (viral load)
HCV: Hepatitis C virus
HCV-Ab: IgG antibody to Hepatitis C virus
HCV-Ag: Hepatitis C virus core antigen
MSM: Men who have sex with men
NAT: Nucleic acid testing
PCR: Polymerase chain reaction (test for viral load)
PPV: Positive predictive value
PWID: People who inject drugs
SDG: Sustainable Development Goals
SVR: Sustained virologic response
WHO: World Health Organisation
